# The short-term impact of *Schistosoma mansoni* infection on health-related quality of life: implications for current elimination policies

**DOI:** 10.1098/rspb.2024.0449

**Published:** 2024-06-12

**Authors:** Sergi Alonso, Moses Arinaitwe, Alon Atuhaire, Andrina Barungi Nankasi, Joaquín M. Prada, Emma McIntosh, Poppy H. L. Lamberton

**Affiliations:** ^1^ School of Biodiversity, One Health and Veterinary Medicine & Wellcome Centre for Integrative Parasitology, University of Glasgow, Glasgow, UK; ^2^ Vector Borne and Neglected Tropical Diseases Control Division, Ministry of Health, Kampala, Uganda; ^3^ Department of Comparative Biomedical Sciences, Faculty of Health & Medical Sciences, University of Surrey, Guildford, UK; ^4^ Health Economics and Health Technology Assessment, School of Health & Wellbeing, University of Glasgow, Glasgow, UK

**Keywords:** neglected tropical disease, schistosomiasis, *Schistosoma mansoni*, health-related quality of life, EQ-5D, Uganda

## Abstract

The WHO aims to eliminate schistosomiasis as a public health problem by 2030. However, standard morbidity measures poorly correlate to infection intensities, hindering disease monitoring and evaluation. This is exacerbated by insufficient evidence on *Schistosoma*’s impact on health-related quality of life (HRQoL). We conducted community-based cross-sectional surveys and parasitological examinations in moderate-to-high *Schistosoma mansoni* endemic communities in Uganda. We calculated parasitic infections and used EQ-5D instruments to estimate and compare HRQoL utilities in these populations. We further employed Tobit/linear regression models to predict HRQoL determinants. Two-thirds of the 560 participants were diagnosed with parasitic infection(s), 49% having *S. mansoni*. No significant negative association was observed between HRQoL and *S. mansoni* infection status/intensity. However, severity of pain urinating (*β* = −0.106; s.e. = 0.043) and body swelling (*β* = −0.326; s.e. = 0.005), increasing age (*β* = −0.016; s.e. = 0.033), reduced socio-economic status (*β* = 0.128; s.e. = 0.032), and being unemployed predicted lower HRQoL. Symptom severity and socio-economic status were better predictors of short-term HRQoL than current *S. mansoni* infection status/intensity. This is key to disentangling the link between infection(s) and short-term health outcomes, and highlights the complexity of correlating current infection(s) with long-term morbidity. Further evidence is needed on long-term schistosomiasis-associated HRQoL, health and economic outcomes to inform the case for upfront investments in schistosomiasis interventions.

## 1. Background

Over 240 million individuals are infected with parasitic helminths called schistosomes, which cause a debilitating neglected tropical disease, schistosomiasis. Millions are forced to live with anaemia and poor nutrition [1,2], particularly young children [3] and over 200 000 are estimated to die from schistosomiasis annually [4]. The life cycle of *Schistosoma mansoni*, which causes intestinal schistosomiasis, passes through humans and freshwater snails, and is driven by inadequate access to safe water, sanitation and hygiene (WASH) due to eggs being excreted in stool and people becoming infected with direct contact with infectious water, facilitating reinfections. This repeated exposure is associated with increased worm burdens and long-term severe disease [5]. It also hampers opportunities by reducing school outcomes and productivity [6,7], limiting the economic growth of endemic communities and their countries [8], which perpetuates the cycle of poverty.

The World Health Organization (WHO) has recommended praziquantel mass drug administration (MDA) as the main control strategy [9], with the initial aim of reducing infection intensities, associated morbidity and disease transmission [9]. The WHO now has an updated goal of schistosomiasis elimination as a public health problem by 2030 [10]. This is defined as reducing the prevalence of heavy infections below 1%, as classified by the number of excreted eggs in stool for *S. mansoni* measured by a single Kato-Katz thick smear (heavy infections: ≥400 eggs per gram of stool (EPG)) [9]. However, available evidence on the association between egg-based infection intensity and morbidity is particularly poor for *S. mansoni* [11], and it remains unclear what the policy implications are for long-term morbidity associated with low-to-moderate infections [12]. This is further exacerbated by the lack of morbidity markers for monitoring and evaluating the effect of infection(s) on disease burden which in turn hampers the robust monitoring and evaluation of intervention outcomes.

There has been a recent call for more accurate measures to develop a robust evidence-based monitoring and evaluation framework for schistosomiasis [12]. The lack of high-quality health indicators associated with infections and the absence of epidemiological models linking short- and long-term morbidity limit the evaluation of health interventions [5,11,13]. This is evidenced by an absence of published cost-effectiveness evaluations for schistosomiasis control interventions [14], which significantly limits the crucial evidence base that decision-making processes depend upon for arguing the case for prioritization of upfront investments in WASH infrastructure or other control interventions aimed at reducing disease burden. Evidence-based investment decisions across different disease areas (questions of allocative efficiency) cannot be made due to the lack of comparable metrics. Disability-adjusted life-years (DALYs) are measures that ignore differentials in resource availability, making them unsuitable for resource allocation and have been criticized by how disability weights are assessed by a group of experts and not by individual-level data [15]. Preference-based health-related quality of life (HRQoL) is the current standard for measuring the quality and quantity of life lived. HRQoL data are measurable at an individual level, providing an understanding of how infections such as *S. mansoni* affect individuals and communities, and also providing a baseline from which to monitor individual- and community-level improvements with treatment and other interventions. Crucially, they also provide data to inform cost–utility analysis of interventions across competing health-related issues. However, HRQoL data associated with *S. mansoni* infection are lacking worldwide and particularly within the sub-Saharan Africa context, despite 85% of schistosomiasis cases occurring in this region [9]. The effect of severe and chronic *S. mansoni* infection has been associated with increased morbidity and reduced HRQoL [16,17]. However, short-term effects of mild infections on HRQoL have yielded contradictory results: some studies showed negative associations among adults and children, with higher impact on emotional rather than physical dimensions [18–20], but others reported no associations [21–23]. Importantly, the effect of MDA on morbidity outcomes and HRQoL remains unclear [20,21,24].

In Uganda, prevalence of schistosomes and soil-transmitted helminths (STH) are unequally distributed, but they remain prevalent nationwide, with district-level estimates ranging from 1.2% to 98% [25,26]. They are often co-endemic, mainly due to their mutual link with inadequate sanitation and poverty, coexisting especially with malaria among other life-threatening diseases. However, the effect of such co-infections on morbidity remains understudied [27]. A recent literature review concluded that the prevalence of malaria–helminth co-infection was high among children, and the impact of co-infections on disease severity and anaemia depended on individual immune responses [28]. Importantly, the interaction of malaria–*S. mansoni* increases malaria incidence and worsens the clinical manifestation of both diseases, likely impacting HRQoL [29].

While long-term heavy *S. mansoni* infections are known to lead to more severe disease, understanding how current infection levels relate to an individual’s existing health state remains poor. The overarching aim of this study was to estimate the short-term impact of *S. mansoni* infection, and its common co-infections, on HRQoL among individuals living in different schistosomiasis-endemic communities in Uganda, with the presence of malaria and STH. Box 1 outlines the study research question and hypotheses. In particular, we (i) described the socio-demographic characteristics and infection status of participating communities; (ii) characterized the health states, self-rated health and HRQoL utilities of individuals living in *S. mansoni* endemic communities; (iii) compared the HRQoL of individuals living in two communities with different *S. mansoni* endemicity levels; and (iv) estimated the determinants of HRQoL in these communities, controlling for health and socio-economic characteristics. The ultimate objective of this study was to provide country-driven evidence that disentangles the relationship between infection and short-term HRQoL, which can be used to inform policy makers’ investment decisions.

Box 1.
**Study main research question and hypotheses**

Published literature containing evidence of the impact of *S. mansoni* infections on HRQoL is scarce and inconsistent in sub-Saharan African communities, despite >85% of schistosomiasis cases found there. We formulated the following research question and hypotheses with the objective to test them in rural Ugandan settings.

*Research question:* Are *S. mansoni* infection, infection intensity or co-infections predictors of short-term HRQoL utility scores in these settings?

We used the ‘short-term’ impact of infection(s) on HRQoL to measure the associations between contemporary infection(s) and associated morbidity on HRQoL, mainly due to *S. mansoni* and frequent co-infections.

*Hypotheses:*

#1. Individuals in the *S. mansoni* high-endemic communities report lower HRQoL compared to lower endemicity communities.

#2. In *S. mansoni* endemic communities, individuals report more problems concerning ‘pain/discomfort’ and ‘anxiety/depression’ dimensions due to the long-term exposure to schistosomiasis and soil-transmitted helminthiases than the Ugandan average.

#3. In *S. mansoni* endemic communities, individuals report lower HRQoL and self-reported health scores than the Ugandan average.

#4. Morbidities associated with contemporary *S. mansoni* infection and co-infections, especially with malaria, predict reduced HRQoL scores.

## 2. Methods

### (a) Study areas

The study was conducted in communities of Mayuge and Tororo districts (eastern Uganda), where part of the team has been working for over 15 years [30]. Mayuge and Tororo are semirural districts of around 470 000 and 517 000 inhabitants, respectively (2014 census estimate), with underdeveloped infrastructure and inadequate access to improved WASH [30]. Both districts are highly endemic for malaria, with a *Plasmodium falciparum* parasite rate above 20%, and several neglected tropical diseases [27,31]. Prevalence of *S. mansoni* and STH infections are highly focal, with a range of infection intensities found within both districts [32]. To account for long-term differences in disease exposure, we chose *S. mansoni* high-endemicity communities in Mayuge and moderate-endemicity communities in Tororo. The WHO classifies schistosomiasis endemicity according to the prevalence among school-aged children based on a single Kato-Katz thick smear: low (<10%), moderate (between 10% and 50%) and high (≥50%) [33].

The WHO-recommended control measures in these communities are as follows: annual community-based MDA for schistosomiasis and STH in high-endemicity communities in Mayuge; biennial school-based MDA for schistosomiasis and STH in low-to-moderate endemicity communities in Tororo; and mosquito vector control, artemisinin-combination therapies and integrated community case management for malaria in both districts [27].

### (b) Data collection

This study received ethical clearance from the Vector Control Division Research Ethics Committee (VCDREC/062), Uganda National Council of Science and Technology (UNCST-HS 2193) and the University of Glasgow Medical, Veterinary and Life Sciences Research Ethics Committee (200160068). Fieldwork activities were conducted in December 2021 in Tororo and May 2022 in Mayuge. We recruited children and adults to conduct a health survey and parasitological examinations. Informed consent was given by signature or thumb print, prior to data and sample collection, and was obtained from all recruited adults, from a parent/legal guardian of all recruited children, and informed assent from all children aged 8 and older.

A bespoke questionnaire was used to retrieve participant information for all individuals aged 8 years and upwards, on socio-demographic characteristics, schistosomiasis-related symptoms, previous/current health conditions and HRQoL. Participants were asked to rate the severity of any symptom from 1 to 5 scale (mild to severe) and report their duration in days. Paper questionnaires were used in Tororo and later digitalized. Smartphones were used in Mayuge (ODK software).

After completion of the questionnaire, recruited participants were invited to provide 3 days of samples of stool and urine for examinations, and 1 day of a finger-prick blood sample. The Kato-Katz thick smear technique was used for a visual, quantitative confirmation of *S. mansoni* and STH including *Ascaris lumbricoides*, *Trichuris* and hookworm, and to calculate infection intensity levels, based on 3 days of duplicate smears [34,35]. We calculated the intensity of *S. mansoni* infection according to the WHO classification based on EPG identified by microscopic inspection using Kato-Katz thick smears: no infection (EPG = 0); light (0 < EPG < 100); moderate (100 ≤ EPG < 400); and heavy (EPG ≥ 400) [10]. A point-of-care circulating cathodic antigen test (POC-CCA) was used to detect *S. mansoni* antigens from urine. As a complementary measure of infection prevalence and semi-quantitative intensity, we used the average of G-scores from 3 days of urine samples, to account for the reported test variability and day-to-day antigen variation [36,37]. Two batches of POC-CCA tests were used (210811080 and 211110105), which were both quality controlled as part of a diagnostics-focused study. It was found that the batches were unlikely to have negatively affected infection intensity and prevalence measures and comparisons [38]. Finger-prick blood samples were used for rapid assessments of *Plasmodium* infection and haemoglobin levels. Malaria diagnosis was performed using the Ag **P.f**/**Pan** Malaria Rapid Diagnostic Test (Standard Diagnostic Inc.). SD Malaria Ag **P.f**/**Pan** test detects the presence of histidine-rich protein 2 (HRP2) antigen of *Plasmodium falciparum* and common *Plasmodium* lactate dehydrogenase of *Plasmodium* species in human whole blood. Diagnosis was captured as positive if either or both test bands turned red, otherwise negative. Anaemia was estimated using WHO haemoglobin thresholds [39]. Anthropomorphic measurements were also recorded. In our study, participants were considered infected by helminths if eggs were observed in at least one of the stool samples provided. Following national guidelines, study participants were treated with 40 mg kg^−1^ praziquantel, 400 mg albendazole and dihydroartemisinin/piperaquine, for schistosomiasis, STH and uncomplicated malaria, respectively. Data analyses were performed in Stata v. 16 (StataCorp). Data and code to reproduce the analysis of this study are available at the Enlighten Research Data Repository (University of Glasgow) [40].

### (c) HRQoL instrument

We used the preference-based instrument EQ-5D-5L to describe and quantify health by measuring HRQoL (non-commercial EuroQol registration identification number: 45864) [41]. Respondents were asked to rate their health on five dimensions at the time of the interview: mobility, self-care, usual activities, pain and discomfort, and anxiety and depression. Participants chose across five levels of severity for each dimension, from no problems to extreme problems, and responded to the EuroQol visual analogue scale (EQ-VAS) to self-rate their health between 0 and 100. We used the EQ-5D-5L questionnaire among all study participants and the EQ-5D-Y-5L version (youth) for children [42,43]. As recommended, we only surveyed those 8 years and above [41]. This design ensured continuity and comparability with the use of resources across adult and children’s health interventions, which are the same in the highly endemic areas [44].

The combination of the EQ-5D-5L responses was used to (i) characterize the health states for all participants; (ii) identify the most common health states reported by the sample; and (iii) observe potential clusters within the distribution. We further calculated HRQoL as the utility decrements associated with the health states reported compared with a state of perfect health. For such calculations, we used the parameters of the Ugandan value set recently published [45].

### (d) Hypothesis #1: comparison of HRQoL values across high and moderate *S. mansoni* endemicity communities

A descriptive analysis of the socio-demographic, parasitological and EQ-5D-5L responses data was conducted prior to the hypotheses testing to compare the results from both communities [46]. Analyses were also conducted by age and considering only *S. mansoni* infections. Bar charts and histograms were used to graphically represent EQ-5D-5L responses per dimension for both communities and the distribution of EQ-VAS and HRQoL scores, respectively. The non-parametric Mann–Whitney test was used to investigate our hypothesis and compare the EQ-5D-5L responses and HRQoL scores of both communities. We further estimated the determinants of the EQ-VAS scores as a function of the EQ-5D-5L responses using a linear regression.

### (e) Hypotheses #2 and #3: comparison of EQ-5D responses, HRQoL values and self-reported health scores between *S. mansoni* endemic communities and the Ugandan average

We used population norm data from the Uganda valuation study to compare EQ-5D-5L responses, HRQoL and EQ-VAS scores with those obtained from both endemic communities included in our study. We used the Pearson *χ* ^2^ to test the hypothesis of whether differences in problems reported in EQ-5D-5L responses between the Ugandan population norms and our study were random or due to the long-term *S. mansoni* exposure of the communities. We further used *t*-tests to assess if the means in HRQoL and EQ-VAS scores differed between the Ugandan average and the communities studied.

### (f) Research question and hypothesis #4: *S. mansoni* infection and morbidity as determinants of HRQoL

We calculated the Spearman’s rank correlation coefficient to respond to the research question and identify correlations between HRQoL utility scores and *S. mansoni* infection and other co-infections, among other variables. We further used univariate regression analysis between HRQoL values and such variables to confirm the results. For model specification, we only considered variables with statistically significant correlations at a 10% level and with the direction of the correlation consistent with the literature. We then conducted multiple regression analysis to model the determinants of the HRQoL utility scores, controlling for a set of demographic (age, gender and community), economic (socio-economic status index, education and occupation) and health variables (severity of symptoms and parasitological infection). The variable ‘age’ controlled for the accumulated effects of recurrent infections on HRQoL, while ‘previous health conditions’ controlled for recent or chronic health conditions. A socio-economic status index was constructed using multiple correspondence analyses based on dwelling characteristics and asset ownership of participants: floor, roof and wall materials, number of rooms for sleeping, and mobile, bicycle and radio ownership [47]. We used a Tobit model due to the censored nature of the data and bootstrap methods to calculate robust standard errors [46]. A linear regression model was also employed as less complex models have been found to perform better with the EQ-5D-5L instrument [46,48].

## 3. Results

### (a) Sample characteristics and parasitological findings

**Figure 1.**
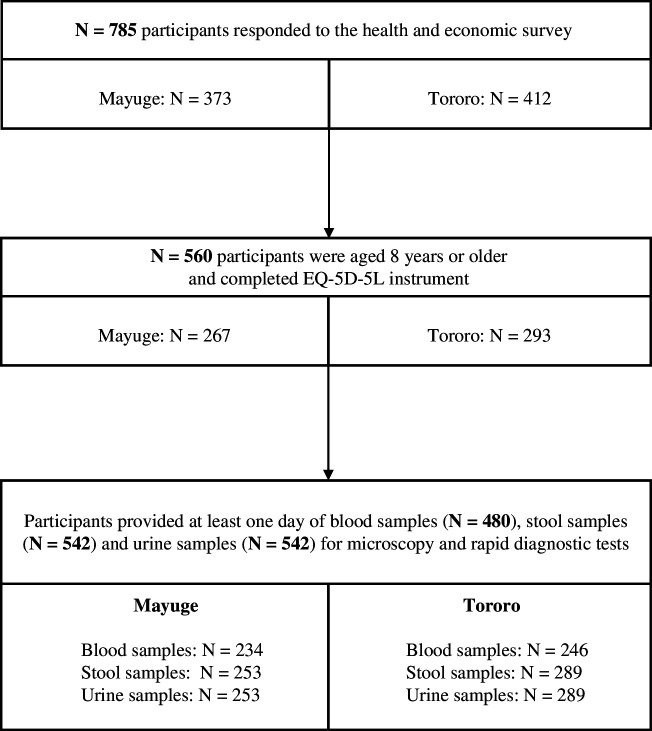
Diagram of participants recruited and human samples obtained for this study. Data were collected in Tororo in December 2021 and in Mayuge in May 2022.

Overall, 785 participants responded to the health community survey (figure 1). The final study sample consisted of 560 participants who were aged 8 years or older at the time of interview and provided complete EQ-5D-5L responses. At least one stool and urine sample were provided by 542 of these respondents and blood samples were provided by 480. Mayuge accounted for 48% of all participants and Tororo 52%.

Adults represented 56% of the study sample and 58% of all participants were females (table 1). A total of 56% of participants were single or unmarried, which was higher in Tororo (61%). Most of the participants reported having achieved up to primary education (76%). The most frequent occupation was agriculture (25%), followed by sales (7%) and fisherfolk (5%). Unemployment was reported by 8% of the sample, but was higher in Tororo (3% versus 13%). Access to water differed across districts: Lake Victoria was the main water source for participants in Mayuge (94%), boreholes with manual pumps (41%) and protected wells (28%) were the main water sources for participants in Tororo.

**Table 1.** Descriptive statistics.

	Mayuge (*n *= 267)		Tororo (*n *= 293)		all (*n *= 560)	
variable	*N*	%	*N*	%	*N*	%
age range (years)						
child (8–17)	113	43.8	131	45.2	244	43.8
adult (≥18)	154	56.2	159	54.8	313	56.2
gender						
female	163	61.3	150	54.4	313	57.7
male	103	38.7	126	45.6	229	42.3
marital status						
single/not living with partner	136	50.9	180	61.4	316	56.4
married/living together	100	37.5	94	32.1	194	34.6
divorced or widowed	31	11.6	19	6.5	50	9
previous health condition reported						
yes	90	34.8	34	11.9	124	22.8
no	160	65.2	251	88.1	420	77.2
education						
none	33	12.4	31	10.6	64	11.4
up to primary	193	72.3	228	77.8	421	75.2
up to secondary or higher	41	15.3	34	11.6	75	13.4
occupation						
unemployed	9	3.3	36	12.3	45	8
student/child	114	42.7	138	47.1	252	45
agriculture	64	24	77	26.3	141	25.2
fisherfolk	24	9	3	1	27	4.8
sales	31	11.6	8	2.7	39	7
household activities	13	4.9	21	7.2	34	6
other	12	4.5	10	3.4	22	4
non-drinking water source						
piped water	9	3.4	14	4.9	23	4.1
protected well	0	0	83	28.7	83	15
unprotected well	2	0.8	47	16.8	49	8.9
borehole with manual pump	1	0.4	118	40.8	119	21.5
lake or other surface water	250	94.3	22	7.6	272	49.1
other	3	1.1	5	1.7	8	1.4

Infection prevalences differed between communities (table 2). A total of 58% of participants of all ages in Mayuge and 41% in Tororo tested positive for *S. mansoni* infection based on up to 3 days of duplicate Kato-Katz thick smears made from stool samples. Prevalences based on POC-CCAs were higher than by Kato-Katz at 62% in Mayuge and 45% in Tororo. One day of Kato-Katz prevalence in school-aged children only, as per the WHO endemicity guidelines, reached 61% in Mayuge and 41% in Tororo, implying that Mayuge’s communities were at high risk for schistosomiasis, while Tororo’s were at moderate risk. Heavy *S. mansoni* infections, as classified by the WHO, represented 20% in Mayuge and 8% in Tororo, while low and moderate infection prevalences were more similar between communities. Mayuge had a lower *Plasmodium* prevalence than Tororo (28% versus 38%), while the opposite was found concerning STH (19% versus 3%). Overall, 69% of the whole sample presented with a detectable parasitic infection, 29% were co-infected with more than one parasite tested for and 16% presented haemoglobin levels below the WHO thresholds for anaemia. Infection intensity classified by age, district and WHO categories can be found in electronic supplementary material, table S1.

**Table 2.** Number of positives for parasitic infection and percentage over tested.

	Mayuge		Tororo		all	
variable	*N*	%	*N*	%	*N*	%
*Schistosoma mansoni* (up to three days of duplicate Kato-Katz)^a^	145	57.54	116	41.28	261	48.97
*Schistosoma mansoni* (POC-CCA, G-score ≥ 3)	154	61.51	125	44.48	279	52.35
*Schistosoma mansoni* (first day of Kato-Katz in school-aged children only—WHO endemicity [33])^a^	57	60.64	49	40.5	NA	NA
infection intensity (schistosomiasis)						
no infection (EPG = 0)	107	42.46	165	57.89	272	50.65
light (EPG > 0 to <100)	50	19.84	55	19.3	105	19.55
moderate (EPG 100 to <400)	44	17.46	41	14.39	85	15.83
heavy (EPG ≥ 400)	51	20.24	24	8.42	75	13.97
*Plasmodium* species (malaria RDT)	64	27.47	91	38.4	155	32.98
soil-transmitted helminth (Kato-Katz)	48	19.12	8	2.85	56	10.35
diagnosed with any parasite tested for (*S. mansoni*, STH or malaria)	174	68.5	195	69.4	369	68.97
co-infected with more than one pathogen	70	27.78	83	29.54	153	28.76
participants with anaemia	34	14.35	41	16.6	75	15.5

Haemoglobin (g /dl) levels to diagnose anaemia were [39]: <13.0 for men >14 years; <12.0 for women >14 years; <12.0 for children 12–14 years; <11.5 for children 8–11 years.

^a^ We found no statistically significant difference between POC-CCA and Kato-Katz measures of prevalence (Pearson’s *χ* ^2^(1) = 1.0645, *p* = 0.30).

EPG, eggs per gram of faeces as measured by up to 3 days of duplicate Kato-Katz thick smears; NA, not applicable; POC-CCA, point-of-care circulating cathodic antigen; RDT, rapid diagnostic test; STH, soil-transmitted helminthiases.

### (b) Result #1: Mayuge and Tororo communities reported similar HRQoL values and health problems, except for the ‘pain/discomfort’ dimension

**Figure 2.**
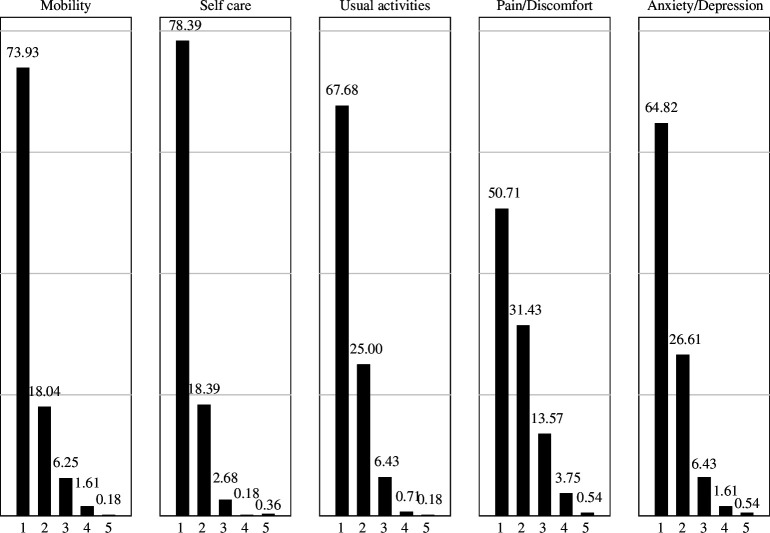
EQ-5D-5L responses by dimension (%). Responses of the 560 participants to the EQ-5D-5L instrument were classified by dimension. For each dimension, participants reported experiencing any problem according to five levels: (1) no problems; (2) slight problems; (3) moderate problems; (4) severe problems; or (5) extreme problems.

A total of 42% of participants reported having ‘no problems’ in any of the five EQ-5D attributes (figure 2 and electronic supplementary material, figure S2), with higher frequency in Tororo (37% versus 47%). However, 49% reported some degree of ‘pain/discomfort’, 35% reported ‘anxiety/depression’ and 22% reported ‘self-care’ problems. Severe to extreme problems were reported by fewer than 5% of the participants. Most of the respondents’ health states consisted of none to slight problems, and to a lesser extent, moderate problems for just one of the five dimensions (levels 1–3). Seventy-one per cent of the sample concentrated on the 10 most common health states, and did not include dimensions with severe or extreme problems (levels 4 and 5) (electronic supplementary material, table S3). The mean score for the EQ-VAS was 72.13% (s.d. = 18.84), but older participants reported statistically significant lower values, which was similar across communities (electronic supplementary material, table S4). The distributions were skewed towards higher values (better health) in both communities (electronic supplementary material, figure S5). When considering all participants, mobility (*β* = −4.80, *p* < 0.05) and ‘pain/discomfort’ (*β* = −4.29, *p* < 0.05) had the biggest impact on the EQ-VAS scores, and ‘anxiety/depression’ the smallest (electronic supplementary material, table S6). However, ‘anxiety/depression’ had the biggest impact when considering only Mayuge (*β* = −7.29, *p* < 0.05). The dimension ‘usual activities’ was not significantly associated with EQ-VAS scores.

Responses on both communities were similar, with the exception of the ‘pain/discomfort’ dimension, where more severe problems were reported in Mayuge than in Tororo (*z* = −2.881, *p* = 0.01) (electronic supplementary material, table S7), also when considering only *S. mansoni* infected individuals (*z* = −2.586, *p* = 0.01) (electronic supplementary material, table S8). However, participants in Tororo reported lower overall HRQoL scores than those in Mayuge (*z* = −1.568, *p* > |*z*| = 0.12), also when considering only *S. mansoni* infections (*z* = −1.693, *p* > |*z*| = 0.09), but the difference was not statistically significant. EQ-VAS values were also similar across communities. Therefore, the hypothesis that participants living in high-endemic communities report lower HRQoL was not supported by our data, except for pain/discomfort (hypothesis #1).

### (c) Result #2: *S. mansoni* endemic communities reported more health problems and lower HRQoL values than the Ugandan average

Participants in our study reported more problems in all dimensions except for ‘anxiety/depression’ problems, which were less reported in our study, compared with population norm data published by the Uganda valuation study [45] (electronic supplementary material, table S9). However, only the dimensions ‘self-care’ (*χ* ^2^(4) = 88.50, *p* < 0.01), ‘usual activities’ (*χ* ^2^(4) = 25.30, *p* < 0.01) and ‘anxiety/depression’ (*χ* ^2^(4) = 10.69, *p* = 0.03) were statistically significantly different compared with the Ugandan average. This implied that the hypothesis that *S. mansoni* communities report more problems due to long-term helminthic exposure was not supported for the dimensions ‘pain/discomfort’ and ‘anxiety/depression’ (hypothesis #2).

HRQoL utility scores estimated for our samples (mean = 0.54, s.d. = 0.89) were statistically significantly lower than the Ugandan average (mean = 0.86, s.d. = 0.20, *t* = −7.92, *p*(|*T*| > |*t*|) < 0.01). EQ-VAS scores were also lower (*t* = −2.68, *p*(|*T*| > |*t*|) = 0.01). Hence, the hypothesis that *S. mansoni* endemic communities report lower HRQoL and EQ-VAS scores than the Ugandan average was supported by our data (hypothesis #3). However, the number of participants reporting perfect health was higher in our study (*t* = −23.25, *p*(|*T*| > |*t*|) < 0.01).

### (d) Result #3: current *S. mansoni* infection, infection intensity or co-infections were unable to predict univariate decreases in short-term HRQoL in moderate and high *S. mansoni* endemic communities

Results from the Spearman’s rank correlation coefficient demonstrated that infection status (*S. mansoni*, malaria, STH), co-infection(s) and intensity of *S. mansoni* infection were not correlated with lower HRQoL values (table 3). Conversely, previous health conditions and severity of schistosomiasis-related symptoms were significantly correlated with decreasing HRQoL utilities. Significant correlations were also identified for specific socio-economic characteristics, such as age, gender, marital status, socio-economic status, education and occupation. The sign and strength of the relationship between HRQoL and the studied variables were confirmed by univariate regression analyses.

**Table 3.** Spearman’s rank correlation coefficient to identify correlations between HRQoL utility scores and socio-demographic, economic and health variables.

variable	*N*	rho	prob > |*t*|
age	**559**	**−0.4284**	**0.0000**
gender	**542**	**−0.1324**	**0.0020**
marital status	**560**	**−0.3964**	**0.0000**
previous health condition (Y/N)	**544**	**−0.1946**	**0.0000**
*S. mansoni* infection (KK)^a^	**542**	**0.1777**	**0.0000**
*S. mansoni* infection (POC-CCA)^a^	**542**	**0.2224**	**0.0000**
*S. mansoni* infection intensity^a^	**546**	**0.1694**	**0.0001**
*Plasmodium* infection^a^	**479**	**0.1963**	**0.0000**
any infection^a^	**544**	**0.1774**	**0.0000**
co-infection^a^	**541**	**0.2147**	**0.0000**
severity of diarrhoea	**550**	**−0.0924**	**0.0303**
severity of blood in stool	555	−0.0609	0.1521
severity of pain urinating	**556**	**−0.2663**	**0.0000**
severity of chills	**550**	**−0.1203**	**0.0047**
severity of headache	**544**	**−0.1213**	**0.0046**
severity of muscle pain	**549**	**−0.3134**	**0.0000**
severity of weakness	**543**	**−0.3020**	**0.0000**
severity of body swelling	**550**	**−0.0779**	**0.0679**
severity of abdominal pain	**550**	**−0.1630**	**0.0001**
socio-economic status index	**560**	**0.0824**	**0.0514**
education	**560**	**0.1035**	**0.0143**
occupation	**560**	**−0.1583**	**0.0002**
EQ-VAS	**551**	**0.5500**	**0.0000**
anaemia	483	−0.0291	0.5241
source of non-drinking water	554	−0.0263	0.5360
latrine location at home	547	0.0123	0.7744

Values in bold are significant at 10% significance.

^a^ Significant associations but variables were not included in the regression model due to having opposite sign found in the published literature.

EQ-VAS, EuroQol visual analogue scale; KK, Kato-Katz thick smear technique; POC-CCA, point-of-care circulating cathodic antigen.

### (e) Result #4: morbidity related to schistosomiasis-specific clinical symptoms and socio-economic factors were important predictors of short-term HRQoL in *S. mansoni* endemic communities

HRQoL utility scores were skewed towards perfect health (HRQoL utility = 1) (electronic supplementary material, figure S10), and were non-normally distributed (Shapiro–Wilk test = 12.313, *n* = 561, *p* < 0.001). For the Tobit model, age (*β* = −0.016, *p* = 0.033), severity of pain urinating (*β* = −0.104, *p* = 0.005), and body swelling (*β* = −0.326, *p* = 0.005) were negatively and significantly associated with HRQoL scores (table 4). Conversely, socio-economic status (*β* = 0.128, *p* = 0.032) and being employed as compared with unemployed were positively associated with HRQoL scores. Endemicity level captured by a geographical variable (Mayuge or Tororo), education achievement and gender were not statistically significant predictors of HRQoL scores in these communities. A multivariate linear regression was able to capture additional symptoms as predictors of lower HRQoL: severity of muscle pain (*β* = −0.002, *p* < 0.001) and severity of headache (*β* = −0.057, *p* < 0.021). Results from multivariate regression analyses supported the hypothesis that morbidity associated with *S. mansoni* and co-infections was associated with lower HRQoL (hypothesis #4).

**Table 4.** Determinants of HRQoL scores.

variable	Tobit regression			linear regression		
	coefficient	s.e.	*p* > |*z*|	coefficient	s.e.	*p* > |*t*|
age	**−0.016**	**0.008**	**0.033**	**−0.011**	**0.003**	**0.004**
gender (base: female)	−0.058	0.131	0.657	−0.004	0.084	0.959
marital status (base: single)						
married/living together	−0.300	0.242	0.214	−0.003	0.137	0.985
divorced/widowed	−0.499	0.452	0.270	−0.286	0.193	0.139
previous health condition (yes/no)	−0.048	0.141	0.737	0.108	0.103	0.292
severity of diarrhoea	−0.081	0.050	0.111	−0.051	0.032	0.107
severity of pain urinating	**−0.106**	**0.043**	**0.005**	**−0.064**	**0.025**	**0.009**
severity of chills	−0.052	0.051	0.217	−0.006	0.027	0.817
severity of headache	−0.053	0.044	0.224	**−0.057**	**0.024**	**0.021**
severity of muscle pain	−0.002	0.005	0.720	**−0.002**	**0.000**	**0.000**
severity of weakness	−0.059	0.057	0.299	0.005	0.037	0.882
severity of body swelling	**−0.326**	**0.125**	**0.005**	**−0.281**	**0.061**	**0.000**
severity of abdominal pain	0.001	0.041	0.951	0.007	0.019	0.714
location/community (base: Mayuge)	−0.022	0.124	0.811	−0.035	0.087	0.679
socio-economic status	**0.128**	**0.060**	**0.032**	**0.103**	**0.049**	**0.034**
education (base: no education) primary secondary or higher	0.285 0.037	0.216 0.240	0.126 0.865	0.158 0.037	0.141 0.167	0.264 0.826
occupation (base: unemployed)						
student	**0.819**	**0.343**	**0.017**	**0.620**	**0.192**	**<0.00**
farmer	**0.852**	**0.360**	**0.018**	**0.726**	**0.156**	**<0.001**
fisherfolk	**1.142**	**0.407**	**0.005**	**0.869**	**0.242**	**<0.001**
sales	0.667	0.347	0.055	**0.651**	**0.208**	**0.002**
household activities	0.792	0.431	0.066	**0.645**	**0.204**	**0.002**
other	**1.225**	**0.376**	**0.001**	**1.026**	**0.235**	**<0.001**
intercept	1.259	0.481	0.086	0.357	0.347	0.304

Tobit regression with bootstrapped standard errors and linear regression. Tobit regression: *n* = 464, uncensored = 268, right-censored = 169, Wald *χ* ^2^ (22) = 128.60, Prob > *χ* ^2^ < 0.001, Pseudo *R* ^2^ = 0.1412. Linear regression: *n* = 464, *F*(22, 441) = 8.24, Prob > *F* < 0.001, *R* ^2^= 0.2857, Adj. *R* ^2^ = 0.2559, Root MSE = 0.81578. Values in bold are significant at 5% significance.

^a^ Symptom associated with schistosomiasis caused by *S. haematobium*.

s.e., standard error.

## 4. Discussion

Current *S. mansoni* infection intensities are poor predictors of available morbidity markers [11]. As WHO strives for the elimination of schistosomiasis as a public health problem, we urgently need improved disease-specific markers and health outcomes, as well as generic preference-based data, such as HRQoL, to inform cost–utility analysis of interventions across competing health-related issues. We quantified the short-term effect of *S. mansoni* and common co-infections on HRQoL among individuals living in moderate and high *S. mansoni* endemicity communities and found that neither current infection status nor intensity were predictors of reduced HRQoL utility scores. Our study, however, is the first to show that the severity of pain, discomfort and inflammation, symptoms commonly associated with *S. mansoni* infection and other co-infections, are important predictors of HRQoL in moderate- and high-endemicity communities in Uganda.

HRQoL decreased with age and was statistically significantly influenced by the socio-economic and employment status of participants, supporting previous studies [18,22]. Higher intensities of current *S. mansoni* infection were unable to predict reduced HRQoL utility scores, implying that morbidity, and likely multimorbidity, is complex and current severity of associated symptoms might be more relevant to understand HRQoL than the current infection status. A positive, or lack of association, between *S. mansoni* infection and HRQoL has previously been reported [21–23]. This is likely explained by delays from infection peaks and actual disease manifestations [5,11]. The majority of schistosomiasis morbidity is caused by the host’s response to eggs trapped in host tissues, with inflammation and granulomas building up over time with long-term chronic infections [5]. As current infection levels do not predict current morbidity levels, it is therefore potentially unsurprising that they are unable to predict short-term HRQoL. However, actual morbidities were negatively associated with HRQoLs. Ultimately, the pathway from infection intensity to morbidity and subsequent HRQoL decrements remains unclear: even light-intensity infections contribute to short-term and long-term schistosomiasis-specific disability [49], which raises concerns about current WHO elimination goals focusing only on reducing heavy infections [10]. Our study further highlights the knowledge and diagnostic gap for monitoring progress towards actually eliminating public health problems associated with schistosomiasis. HRQoL scores are likely unsuitable in their current form or used in isolation. Due to the time delay between infection and morbidity peaks a new model, such as those used for smoking, for example, may hold some of the answers, where current prevalence does not predict long-term risk [50]. However, unlike smoking where previous exposure can be recalled more easily, schistosomiasis history is harder to identify. We attempted to quantify this by conducting HRQoL surveys in communities with different *S. mansoni* endemicity, hypothesizing that highly endemic communities will have individuals with heavier and chronic infections. In Mayuge, we found evidence for increased ‘pain/discomfort’ in comparison to those in Tororo, which may be due to previous and/or continuous *S. mansoni* exposure. However, further studies are required to confirm causality between longer disease exposure and ‘pain/discomfort’. Multifactorial approaches and frameworks enabling a broader societal perspective based on observable outcomes, such as productivity losses and school attendance/performance, should also be explored to justify large upfront investments in the prevention and control of schistosomiasis, given the large impact on health and economic opportunities the disease inflicts in these endemic communities.

Uganda was the first country in sub-Saharan Africa to implement MDA with praziquantel in 2003. However, in 2016 treatment coverage in Mayuge reached only 45% of all eligible residents with 35% reported having never taken the drug, the latter likely acting as reservoirs for the reinfection of treated individuals, alongside environmental reservoirs [51]. Low praziquantel coverage in the district has been reported as early as in 2009, and might be one of the main reasons why prevalence rates remain above 50% in some parts of Mayuge [52]. Consequently, parasitological data in our study confirmed that almost half of the study participants presented with a detectable *S. mansoni* infection, more than two-thirds presented at least one parasitic infection and almost one-third were co-infected with at least two different parasites. Nevertheless, participants reported relatively high-valued health profiles, including 42% reporting perfect health (no problems for any dimension). This further demonstrates the complexity of *S. mansoni* and co-infections in endemic communities, where individuals might be used to living with recurrent infections and potentially the morbidity that they cause from an early age. Surprisingly, participants with malaria, or presenting *S. mansoni* co-infections, reported higher HRQoL values than those uninfected. Fürst *et al*. and Hürlimann *et al*. [18,22], also found that participants with malaria reported higher HRQoL score on average. Continuous exposure to malaria generates naturally acquired immunity, while most cases were uncomplicated, the effect of (co-)infections on morbidity and subsequent HRQoL remains inconclusive. Further studies are needed, using longitudinal epidemiological data, to disentangle the link between infection(s) and morbidity, and how recurrent infections and co-infections influence severe disease progression [28].

Finally, participants in our study reported more problems than most of the population norms in 20 upper-, middle- and high-income countries, except for the dimension ‘anxiety/depression’, and the average EQ-VAS score was among the lowest of the 70.4–83.3 range in those countries [53]. However, this comparison should be made with caution, as the Ugandan population is young and schistosomiasis and other infectious diseases are more prevalent in school-aged children than in adults, with children more heavily infected [54,55], but reporting fewer severe symptoms [11] and higher HRQoL scores [56]. Similarly, reported health and HRQoL scores from the moderate and high *S. mansoni* endemic communities were lower than the Ugandan average. Unexpectedly, however, we found no differences in overall HRQoL across both communities, even when considering only individuals with confirmed *S. mansoni* infection. This effect is challenging to interpret and even though high-endemicity communities for schistosomiasis might be subject to more morbidity [57], genetic factors and predisposition for infection(s) might be more important than endemicity levels.

### 4.1. Limitations

Despite the widespread use of the EQ-5D instruments, cultural, personal lived experiences and/or language issues associated with its use in rural and isolated communities in sub-Saharan Africa may limit the capacity of individuals to self-assess their HRQoL using this instrument [58]. Individuals might not perceive reduced HRQoL for several reasons. First, they are exposed to multiple different endemic diseases and constant reinfections since birth, causing multiple but often overlapping health issues. Second, habituation to mild infections might explain how participants under-report problems regarding ‘anxiety/depression’ and potentially other dimensions as continuous disease exposure reduces their level of response to health issues as they might not know what it feels like to be fully healthy [59]. Third, social stigma and health taboos might have also influenced participants’ responses in either direction [45,60], for example, with such high proportions of infected and co-infected, individuals might likely feel they are ‘no worse off’ than others, resulting in an under-reporting of HRQoL. This highlights potential flaws with the EQ-5D instruments as a measure of HRQoL associated with infectious diseases in these settings, thus underscoring the need to develop new methods to measure HRQoL or to measure societal indicators (productivity/school outcomes) as part of cost–consequence analyses, as recommended by current public health economic evaluation guidance [61]. Finally, our study might be subject to selection bias, given that part of the recruitment was performed at schools, potentially recruiting more school-attending children of specific ages than non-attenders or absent children. Children attending schools might be healthier than absent children, thus under-reporting any health issues, while families of non-enrolled children may have lower socio-economic determinants, already known to negatively affect HRQoL [56]. However, to minimize this impact, efforts were made to recruit children and adults at their homes, with most children in our sample recruited this way, likely minimizing these biases.

## 5. Conclusion

Our study identified important economic- and *Schistosoma mansoni*-related symptoms as predictors of short-term HRQoL, while illustrating the complexity and knowledge gaps on the effects of *S. mansoni* and its common co-infections on disease severity and HRQoL. Short-term HRQoL scores cannot be used to accurately reflect the effect of current schistosomiasis. Further research is needed to understand the mechanisms associated with *S. mansoni* infection and their effects on health outcomes and HRQoL along different time horizons. Until such methodological limitations are overcome, investments in the prevention and treatment of neglected tropical diseases such as schistosomiasis will be hampered.

## Data Availability

Data and code to reproduce the analysis of this study are available at the Enlighten Research Data Repository (University of Glasgow) [62]. Supplementary material is available online [63].
